# Peer victimisation during adolescence and its impact on depression in early adulthood: prospective cohort study in the United Kingdom

**DOI:** 10.1136/bmj.h2469

**Published:** 2015-06-03

**Authors:** Lucy Bowes, Carol Joinson, Dieter Wolke, Glyn Lewis

**Affiliations:** 1 Department of Experimental Psychology, University of Oxford, Oxford OX1 3UD, UK; 2 Centre for Academic Mental Health, School of Social and Community Medicine, University of Bristol, Bristol, UK; 3 Department of Psychology and Division of Mental Health and Wellbeing, University of Warwick, Coventry, UK; 4 Division of Psychiatry, Faculty of Brain Sciences, University College London, London, UK

## Abstract

**Objective** To investigate the strength of the association between victimisation by peers at age 13 years and depression at 18 years.

**Design** Longitudinal observational study.

**Setting** Avon Longitudinal Study of Parents and Children, a UK community based birth cohort.

**Participants** 6719 participants who reported on peer victimisation at age 13 years.

**Main outcome measures** Depression defined according to international classification of diseases, 10th revision (ICD-10) criteria, assessed using the clinical interview schedule-revised during clinic assessments with participants when they were aged 18 years. 3898 participants had data on both victimisation by peers at age 13 years and depression at age 18 years.

**Results** Of the 683 participants who reported frequent victimisation at age 13 years, 101 (14.8%) were depressed according to ICD-10 criteria at 18 years; of the 1446 participants reporting some victimisation at age 13 years, 103 (7.1%) were depressed at age 18 years; and of the 1769 participants reporting no victimisation at age 13 years, 98 (5.5%) were depressed at age 18 years. Compared with children who were not victimised those who were frequently victimised by peers had over a twofold increase in odds of depression (odds ratio 2.96, 95% confidence interval 2.21 to 3.97, P<0.001). This association was slightly reduced when adjusting for confounders (2.32, 1.49 to 3.63, P<0.001). The population attributable fraction suggested that 29.2% (95% confidence interval 10.9% to 43.7%) of depression at age 18 years could be explained by peer victimisation if this were a causal relation.

**Conclusion** When using observational data it is impossible to be certain that associations are causal. However, our results are consistent with the hypothesis that victimisation by peers in adolescence is associated with an increase in the risk of developing depression as an adult.

## Introduction

Depression is a leading contributor to the global burden of disease.^1^ The incidence and prevalence of depression increases rapidly from childhood to early adulthood, and by age 18 years the prevalence is similar to that in adults.^2^ This has led to suggestions that school based preventive programmes could help to reduce the burden of depression. However, the results of cognitive behaviour programmes in schools have been disappointing.^3^

Adolescents spend more time with their peers than do children and adults, and peers are used as primary sources for social comparison and appraisal.^4^ Victimisation by peers or “bullying” has been proposed as one potentially modifiable risk factor for depression. Cross sectional studies report correlations between peer victimisation and clinical depression,^5^ ^6^ whereas adults with depression are more likely to retrospectively report peer victimisation as a child or adolescent^7^; however, these results could be due to recall bias or reverse causality. Previous longitudinal studies have mainly examined outcomes such as internalising symptoms in childhood or adolescence^8^ ^9^ ^10^ rather than studying diagnoses of depression at older ages, which has more relevance for the population burden of depression that occurs mainly in adults of working age.^11^ ^12^

We are only aware of a small number of longitudinal studies that have prospectively investigated victimisation in relation to depression meeting diagnostic criteria in late adolescence or adulthood. Some of these did not adjust for key confounders such as baseline depressive symptoms^13^ or behavioural problems,^14^ which might themselves have led to both victimisation and later depression. Others^15^ ^16^ have only found an association for children who were both victims and perpetrators of bullying. Perpetrators also display high levels of conduct problems and reactive aggression, which are themselves associated with later depression^17^ ^18^ and may confound the association. Others have not used a well validated measure of bullying, relying on reports from single items.^14^ ^15^ ^16^ ^19^ Given the limitations of previous studies, it still remains unclear whether peer victimisation during adolescence contributes to the public health burden of depression that mostly manifests in adulthood.

Using data from over 3700 families from the Avon Longitudinal Study of Parents and Children (ALSPAC) cohort in the United Kingdom, we investigated the strength of the association between being victimised by peers in early adolescence and the emergence of depression at age 18 years, while taking account of several confounders, including previous victimisation in childhood and mental health problems. All participants with data on peer victimisation at age 13 years and at least one assessment of depressive symptoms were eligible to be included in the study. Our hypothesis was that peer victimisation during adolescence would be associated with an increased risk of developing depression at age 18 years. We also estimated the incidence of depression that might be attributable to peer victimisation in adolescence if this relation was causal.

## Methods

### Sample

The sample comprised participants from the ALSPAC cohort (www.alspac.bris.ac.uk)^20^; ALSPAC is a transgenerational prospective observational study investigating a wide range of influences on the health and development of children. All pregnant women resident in the former Avon Health Authority in south west England, with an estimated date of delivery between 1 April 1991 and 31 December 1992, were eligible to take part. Women were recruited as early as possible in their pregnancy, resulting in a cohort of 14 541 pregnancies and 13 988 children alive at 12 months of age. When the oldest children were aged 7 years, we attempted to increase the size of the initial sample with eligible cases that did not join the cohort at the outset. The phases of enrolment are described in more detail in the cohort profile paper.^21^ In the current study we use data from the subsample of ALSPAC who attended the most recent research clinic for the children. 

### Depression

Participants completed a self administered computerised version of the clinical interview schedule-revised^22^ at the 18 year research clinic (mean age 17 years 10 months). This schedule enables diagnoses for common mental disorders to be derived from the international statistical classification of diseases, 10th revision (ICD-10). Participants were asked about symptoms of depression in the past one week to one month (depending on the symptom). In addition, participants were asked the question: “How long have you been feeling sad, miserable, or depressed, or unable to enjoy or take an interest in things you have described?,” responding “less than two weeks” (n=507), “two weeks to six months” (n=525), “six months to one year” (n=194), “one to two years” (n=100), or “two or more years” (n=87).

### Peer victimisation

We assessed peer victimisation through self report using a modified version of the bullying and friendship interview schedule.^23^ The items are listed in table 1. Participants were asked whether they had experienced nine different types of peer victimisation in the past six months, responding “no,” “yes sometimes” (<4 times), “yes repeatedly” (≥4 times), or “yes very frequently” (at least once per week). The items related to both relational and overt victimisation (for example, exclusion by peers, lies or nasty things said about them, personal belongings taken, threatened or blackmailed, hit or beaten up). In preliminary analyses we found that scores for relational and overt victimisation were strongly correlated (r=0.61). Furthermore, we did not believe that separating the two broad types of victimisation would substantially add to the policy implications of our study, and so we used an overall measure of peer victimisation.

The victimisation variable was created from the sum of all questions relating to victimisation. Children scored 0 if they had never been bullied. The range of scores was 0-25 (mean 1.85, SD 2.78) and Cronbach’s α was 0.73. To investigate a possible dose-response relation between victimisation and depression, we also created a three level ordinal variable for victimisation.^24^ Children who were never victimised (n=3090) scored 0, children who were occasionally victimised (n=2430) scored 1-3 and were coded as 1, and children who were frequently victimised (n=1199) scored 4 or more and were coded as 2.

Peer victimisation was also reported by mothers when their children were 12 years of age. During questionnaire assessments the mothers were asked whether their offspring was often picked on or bullied by other children,^25^ responding not true (n=5522, 80.5%), somewhat true (n=1108, 16.2%), or certainly true (n=229, 3.3%). The inter-rater agreement between mother and self reports of peer victimisation was low (κ=0.10), similar to that in other studies.^26^

#### Peer victimisation at ages 8 and 10

Peer victimisation was also assessed through interviews with the children at ages 8 and 10 years, using the same version of the bullying and friendship interview schedule. Our measure of victimisation at each time point was created using the same procedure as described for age 13 years. Cronbach’s α was 0.77 and 0.70, respectively.

The polychoric correlations for peer victimisation at age 13 years was 0.29 (SE 0.02) for peer victimisation at age 8 years and 0.40 (SE 0.01) for peer victimisation at age 10 years.

### Confounders

#### Individual characteristics of study children

We assessed the children’s emotional and behavioural problems using maternal reports from the strengths and difficulties questionnaire,^25^ when the children were aged 7 years. Concurrent depressive mood was assessed using the self reported short moods and feelings questionnaire,^27^ when the children were aged 13 years. Perpetration of concurrent bullying at age 13 was assessed using the modified version of the bullying and friendship interview schedule.^23^ As with the victimisation variable, we created the bullying variable from the sum of all questions relating to perpetration of bullying. Children scored 0 if they had never bullied another child (range 0-25).

#### Family characteristics

We adjusted for the effects of several family characteristics, including highest maternal education (dichotomised to advanced level qualifications, university degree, ordinary level qualifications versus certificate of secondary school education, vocational, none); parental occupational social class (derived as the lower of either maternal or paternal social class and dichotomised into non-manual and manual work),^28^ maternal depression (Edinburgh postnatal maternal depression scale^29^), and child maltreatment (no or present) when the study child was aged 7 years, assessed using maternal reports of the study children’s exposure to stressful life events between 5 and 7 years of age. A score of 1 was coded if parents responded yes to any item relating to physical or sexual abuse, or reported that the study child had been put into care.

### Statistical analyses

In univariable models we used logistic regression analyses to calculate odds ratios for depression at age 18 according to victimisation at age 13 (measured as a three level categorical victimisation variable and as a continuous variable). A quadratic term was used to investigate the possibility of a non-linear relation between victimisation and depression. We conducted a final multivariable model including all confounding variables. To investigate whether any effects of victimisation as an adolescent and victimisation as a child (assessed at ages 8 and 10 years) were independent of each other, we examined a series of logistic regression models, and then included all three time points of victimisation into a multivariable model.

We used the punaf command available in STATA 12 to calculate the population attributable risk and 95% confidence interval from the final multivariable logistic regression model.

### Missing data

A sample with complete data across all exposure, outcome, and confounding variables (n=2668) was used to investigate the main and independent effects of victimisation by peers in adolescence. In ALSPAC there is a wealth of information on sociodemographic variables and other variables that predict missingness. These data therefore make the “missing at random” assumption underlying multiple imputation much more reasonable. We could also use previous reports of depressive symptoms to impute ICD-10 depressive disorder at age 18 up to a sample size of 6472.

We used multivariate imputation by fully conditional specification using chained equations (MICE) in Stata 12.^30^ Multiple imputation was conducted in two stages: imputing missing confounders to give a sample size of 3898, then imputing on outcome to give a sample size of 6472. The imputation model included 18 variables in addition to those included in the analyses that were associated with either missingness or depression at age 18 years. These included maternal age and family adversity, and sociodemographics in pregnancy and early childhood (the full list is available on request). We used Rubin rules to average the variable estimates over 60 imputed or completed datasets.^31^

## Results

Data on victimisation by peers at age 13 years were available for 6719 participants. Of these, 3898 completed the clinical interview schedule-revised at age 18 years. In total, 2668 participants had complete data on all variables, including confounders (figure[Fig fig1]). Attrition depended on several factors, but those lost to follow-up were no more likely to have been bullied as an adolescent (odds ratio 1.00, 95% confidence interval 0.98 to 1.02, P=0.78) than those with complete data.

**Figure fig1:**
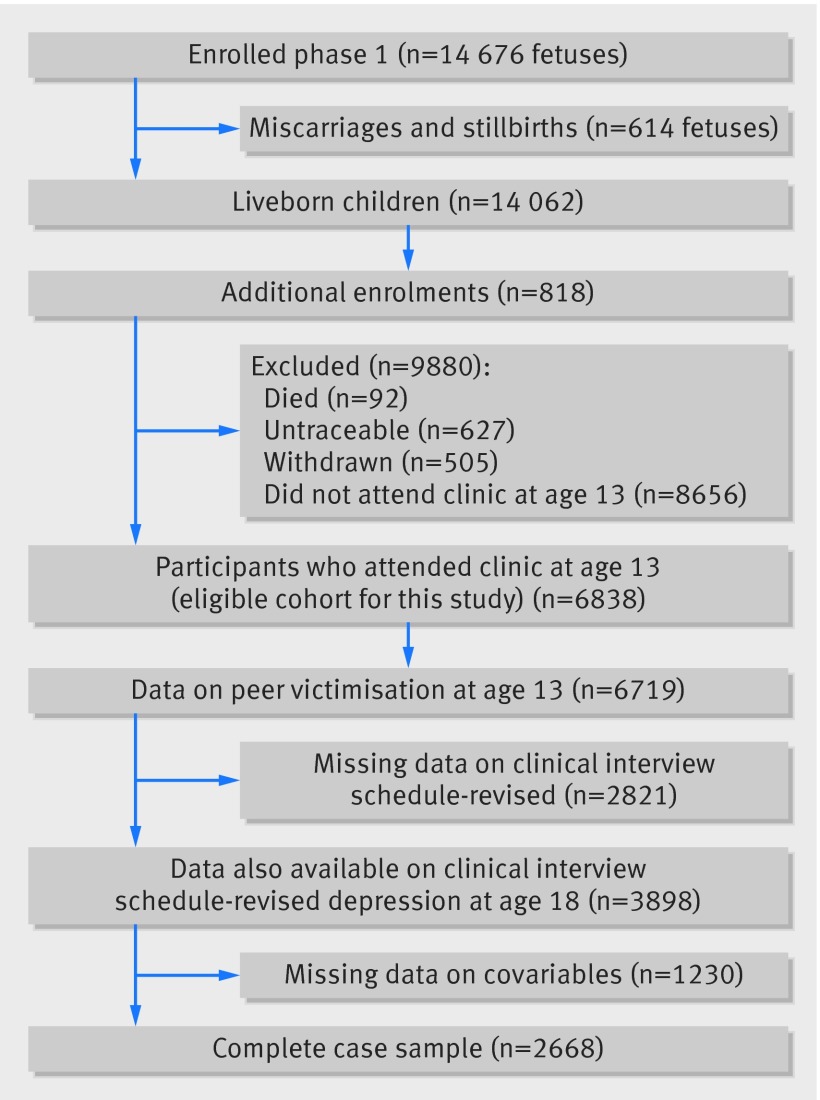
Flowchart of participants in Avon Longitudinal Study of Parents and Children

Table 1 shows the frequencies of victimisation experiences and percentage of children who did not tell anyone at home or the teacher at school. The most commonly reported type of victimisation experience was being called nasty names or having personal belongings taken. Most teenagers (41-74%) reported that they never told their teachers about their victimisation experiences, and 24-51% of teenagers reported that they never told their parents. Teenagers were most likely to report physical victimisation such as being hit or beaten up.

**Table 1 tbl1:** Frequencies of victimisation experiences and percentage of children who did not tell anyone at home or the teacher at school. Values are numbers (percentages) unless stated otherwise

Items	Frequency of victimisation					Not reporting victimisation (%)	
	Never*	Occasional* (1-3 times)	Frequently* (>4 times)	Very frequently (>1/week)		No one told at home	Teachers not told
Someone took personal belongings	5240 (77.6)	1113 (16.5)	234 (3.5)	168 (2.5)		32.8	57.6
Someone threatened or blackmailed teenager	6116 (90.5)	478 (7.1)	105 (1.6)	56 (0.8)		28.2	48.5
Someone hit or beat up teenager	5984 (88.6)	582 (8.6)	120 (1.8)	68 (1.0)		24.8	41.0
Someone tricked teenager	6234 (92.2)	446 (6.6)	46 (0.7)	35 (0.5)		30.2	54.5
Someone called teenager nasty names	4319 (64.0)	1243 (18.4)	598 (8.9)	584 (8.7)		34.8	51.9
Peers would not hang around just to upset teenager	6056 (89.8)	490 (7.3)	126 (1.9)	69 (1.0)		28.5	74.3
Peers tried to get teenager to do things he or she did not want to do	6193 (92.0)	423 (6.3)	77 (1.1)	41 (0.6)		50.8	64.1
Peers told lies about teenager	5608 (83.7)	765 (11.4)	221 (3.3)	105 (1.6)		34.3	66.5
Peers spoilt games to upset teenager	6415 (95.2)	230 (3.4)	51 (0.8)	41 (0.6)		44.4	62.1

*In past six months.

Table 2 shows the characteristics of our complete case sample versus the overall ALSPAC sample. Compared with the overall ALSPAC sample, participants retained in the complete case sample were more likely to be female and to have higher mean levels of emotional problems at age 10 years by maternal report. They were less likely to report concurrent perpetration of bullying and to have parents with lower social class or mothers with lower levels of education. Their mothers had fewer depressive symptoms compared with the ALSPAC sample. There were no differences between the complete case sample and the overall ALSPAC sample in concurrent depressive symptoms at age 13 years, in peer victimisation at age 13 years, or in depression at age 18 years.

**Table 2 tbl2:** Characteristics of complete case sample and overall Avon Longitudinal Study of Parents and Children (ALSPAC). Values are means (standard deviations) unless stated otherwise

Characteristics	Samples		Total No available	P value
	Complete case (n=2668)	ALSPAC*		
Individual characteristics:				
No (%) male	45.5	53.1	13 615	<0.001
Emotional problems (10 years)	1.48 (1.40)	1.68 (1.50)	8244	<0.001
Conduct problems (10 years)	1.49 (1.65)	1.52 (1.68)	8234	0.53
Concurrent depressive symptoms (13 years) (MFQ)	3.91 (3.77)	3.99 (3.88)	6188	0.46
Concurrent bullying perpetration (13 years)	0.81 (1.69)	0.97 (2.11)	6172	0.002
Family characteristics:				
Lower parental social class (%)	40.4	57.8	10 282	<0.001
Maternal education O level or less (%)	48.4	66.6	11 355	<0.001
Mean (SD) maternal depression	6.07 (4.40)	7.23 (4.95)	11.660	<0.001
Maltreatment (≤9 years) (%)	3.0	3.3	8260	0.48
Mean (SD) peer victimisation score at 13 years	1.85 (2.69)	1.87 (2.87)	6211	0.78
Depression at age 18, based on clinical interview schedule-revised (%)	7.4	8.7	4197	0.12

MFQ=short moods and feelings questionnaire.

Percentages are based on proportion of those with data on these variables, as indicated in “Total No” column.

*For core singleton ALSPAC sample not in complete case sample, n=10 949.

Table 3 shows the sociodemographic and other characteristics of children who were never, occasionally, or frequently victimised at age 13 years. Compared with those who were not victimised, adolescents who were victimised by peers at age 13 years were more likely to be female and more likely to have displayed higher levels of emotional and behavioural problems before being bullied. Victimised adolescents were also more likely to display higher levels of concurrent depressive symptoms.

**Table 3 tbl3:** Sociodemographic and other characteristics of participants, by peer victimisation at age 13 years. Values are means (standard deviations) unless stated otherwise

Characteristics	No victimisation (n=3090)	Occasional victimisation (n=2430)	Frequent victimisation (n=1199)	P value
Individual characteristics:				
Male (%)	50.6	47.3	47.6	0.02
Emotional problems	1.3 (1·6)	1.4 (1·7)	1.8 (1·9)	<0.001
Conduct problems	1.1 (1·3)	1.2 (1·3)	1.5 (1·6)	<0.001
Concurrent depressive symptoms (MFQ)	1.0 (0.7)	1.4 (0.7)	1.9 (0.7)	<0.001
Concurrent bullying perpetration (13 years)	0.2 (0.9)	1.0 (1.8)	2.5 (2.9)	<0.001
Family characteristics				
Lower parental social class, (%)	47.7	44.6	46.0	0.12
Maternal education: O levels or less (%)	57.2	53.7	53.1	0.01
Maternal depression	6.2 (4.4)	6.6 (4.6)	6.9 (4.9)	<0.001
Maltreatment (%)	2.0	3.1	4.9	<0.001

MFQ=short moods and feelings questionnaire.

### Association with depression

The proportion with depression increased with the frequency of victimisation (table 4). Just over 5% of teenagers in the no victimisation group had depression increasing to nearly 15% in those reporting frequent victimisation. In terms of persistent depression, 10.1% of those who reported frequent victimisation said that they had experienced depressive symptoms for more than two years, compared with 4.1% of those in the non-victimised group. Despite a difference in overall prevalence rates between sexes, there was no evidence of an interaction between sex and peer victimisation (P=0.45), and thus analyses were conducted for the whole sample and not stratified by sex.

**Table 4 tbl4:** Odds ratios for ICD-10 depression at age 18 years by victimisation at age 13 years

Victimisation status	No (%) depressed	Unadjusted odds ratio (95% CI)			Odds ratio (95% CI)			
		All available data (n=3898)	Complete cases (n=2668)		Adjusted (n=2668)	Unadjusted using imputed dataset* (n=6472)	Adjusted using imputed dataset† (n=3898)	Adjusted using imputed dataset* (n=6472)
None	1769 (5.5)	1.00	1.00		1.00	1.00	1.00	1.00
Occasional	1446 (7.1)	1.31 (0.98 to 1.74)	1.34 (0.93 to 1.93)		1.08 (0.74 to 1.59)	1.35 (1.01 to 1.81)	1.08 (0.80 to 1.46)	1.13 (1.02 to 1.12)
Frequent	683 (14.8)	2.96 (2.21 to 3.97)	3.33 (2.32 to 4.78)		2.32 (1.49 to 3.63)	2.82 (2.05 to 3.87)	2.00 (1.39 to 2.87)	1.87 (1.29 to 2.72)
Linear trend	—	1·13 (1·09 to 1·17)	1.14 (1.09 to 1.18)		1.08 (1.02 to 1.14)	1.12 (1.08 to 1.16)	1.07 (1.03 to 1.12)	1.07 (1.02 to 1.12)

Adjustments: children’s individual characteristics (sex, emotional problems, behavioural problems, concurrent depressive symptoms, and concurrent bullying perpetration) and family characteristics (parental social class, mother’s education, maternal depression, and maltreatment as a child).

*Data on confounders and outcome (for those participants with a previous measure of depressive symptoms) imputed (n=6472).

†Only data on confounders imputed (n=3898).

The increased rates of depression in those with frequent victimisation corresponded to an odds ratio of 2.96 (95% confidence interval 2.21 to 3.97) compared with those who were not victimised. This association was reduced slightly when adjusting for confounding factors. The largest reduction in effect size was observed when adjusting for the effects of children’s individual characteristics (sex, baseline emotional and behavioural problems, and concurrent depressive symptoms; odds ratio 2.14, 42% reduction in effect size). Evidence of a dose-response relation was observed between the continuous measure of victimisation and depression (adjusted linear trend odds ratio 1.08, P=0.01). There was little evidence for a non-linear relation between victimisation and depression (P=0.07). We found no evidence of an interaction between depression and peer victimisation at 13 years in predicting depression at 18 years (P=0.34, likelihood ratio test χ^2^=2.19), suggesting that victimisation is associated with both onset and persistence of depression. The population attributable fraction from the final multivariable logistic model suggested that 29.2% (95% confidence interval 10.9% to 43.7%) of the total risk of depression at age 18 could be explained by peer victimisation in adolescence.

### Sensitivity analyses

In additional sensitivity analyses we removed all participants with scores reaching clinical significance on the short moods and feelings questionnaire at age 13 (defined as scores of ≥11; n=473). Our analyses were virtually unchanged: occasional victimisation 1.29 (0.87 to 1.92) and frequent victimisation 2.82 (1.86 to 4.26). Additionally, when the 87 participants who reported depressive symptoms for more than two years (according to the clinical interview schedule-revised) were excluded from the analyses, the results were only slightly attenuated: occasional victimisation 1.17 (0.85 to 1.60) and frequent victimisation 2.20 (1.54 to 3.14).

We also investigated whether mother reports of victimisation of their offspring during adolescence were associated with depression at age 18 years. Mother reports of victimisation at 12 years of age were associated with depression at age 18 years in unadjusted analyses (1.38, 1.10 to 1.74); however, there was no evidence for an association after adjusting for confounders (1.08, 0.78 to 1.49).

Owing to high amounts of missing data, we were unable to adjust for the potential confounding effects of paternal mental health in our multivariate models. In additional analyses, paternal depression and anxiety (assessed using the Crown Crisp experiential index) were associated with victimisation in offspring at age 13 (β=0.02, 95% confidence interval 0.01 to 0.03, P<0.001) and depression in offspring (1.02, 1.00 to 1.04, P=0.05); however, neither attenuated the strength of the unadjusted relation by much (unadjusted odds ratio 1.13, 95% confidence interval 1.09 to 1.17, P<0.001, adjusted for paternal depression and anxiety 1.12, 1.07 to 1.17, P<0.001).

### Missing data analyses

Analyses were repeated using the imputed datasets (table 4). Results were consistent with the previous findings based on complete cases. The associations between overall victimisation score and depression were slightly attenuated—for example, the odds ratio for the fully adjusted model using the linear measure of victimisation was 1.08 (95% confidence interval 1.02 to 1.14) compared with 1.07 (1.02 to 1.12) in the imputed dataset. However, the evidence of an association between victimisation and depression was still strong.

### Victimisation in adolescence versus childhood 

Overall rates of victimisation appeared to decline with age, with mean scores of 3.27 (SD 3.6) at age 8 years, 2.01 (SD 2.8) at age 10, and 1.82 (SD 2.8) at age 13. Correlations across age were modest, with polychoric correlation coefficients between 0.29 and 0.40. Despite the decrease in rates of victimisation, 255 new victims were identified at 13 years of age who had not reported victimisation at ages 8 and 10 years. The univariable results provide evidence for associations between victimisation in childhood (assessed at ages 8 and 10 years) and victimisation in adolescence with depression at age 18 years (table 5). There was still evidence of an association between victimisation at age 13 years and depression when all three exposure time points were included in the regression model.

**Table 5 tbl5:** Odds ratios for ICD-10 depression at age 18 years by victimisation during childhood (ages 8 and 10 years) and adolescence (age 13)

Victimisation status by age	No (%) depressed	Odds ratio (95% CI)		
		Unadjusted	Unadjusted complete case* (n=3139)	Adjusted† (n=3139)
8 years:				
None	980 (6.3)	1.00	1.00	1.00
Occasional	1930 (7.2)	1.16 (0.85 to 1.58)	1.18 (0.85 to 1.64)	1.03 (0.73 to 1.45)
Frequent	708 (11.7)	2.02 (1.43 to 2.87)	1.91 (1.32 to 2.77)	1.38 (0.92 to 2.08)
10 years:				
None	1674 (6.3)	1.00	1.00	1.00
Occasional	1510 (8.2)	1.31 (0.99 to 1.72)	1.33 (0.98 to 1.80)	1.15 (0.84 to 1.57)
Frequent	754 (10.3)	1.68 (1.24 to 2.29)	1.83 (1.30 to 2.58)	1.18 (0.80 to 1.73)
13 years:				
None	1769 (5.5)	1.00	1.00	1.00
Occasional	1446 (7.1)	1.26 (0.94 to 1.68)	1.24 (0.·90 to 1.69)	1.17 (0.85 to 1.62)
Frequent	683 (14.8)	2.88 (2.14 to 3.87)	2.95 (2.13 to 4.08)	2.58 (1.81 to 3.67)

*Complete case variable=data complete for clinical interview schedule-revised, and victimisation at three time points, n=3139.

†Victimisation at all time points entered into model.

## Discussion

We found evidence for an association between victimisation by peers in adolescence and depression in young adulthood. There was a dose-response relation between frequency of peer victimisation in adolescence and the risk of developing depression meeting ICD-10 criteria at age 18 years. Adolescents who reported frequent bullying by peers were about twice as likely to develop depression compared with non-victimised peers, even after adjustment for previous depressive symptoms and previous victimisation and a range of other individual and family confounding factors. This association was seen in both males and females. The large population attributable fraction suggests that approximately 29% of the burden of depression at age 18 years could be attributed to victimisation by peers in adolescence if this were a causal relation. The longitudinal nature of our study reduces the possibility of reverse causality, whereas the impact of victimisation on depression has both clinical and biological plausibility. All these points support the hypothesis that this is a causal relation, although this is difficult to establish using observational data.

### Strengths and weaknesses of this study

This study has several strengths. The large sample size and extended follow-up from early childhood to late adolescence spanned the period in which rates of depression increase rapidly and approach those found in adulthood.^2^ We had detailed self report measures of peer victimisation and adjusted for a large number of potential confounders, including previous victimisation. There is a concern that the victims might have characteristics that increase susceptibility to victimisation as well as independently increasing the risk of depression. With the wealth of previous data available in ALSPAC, our analyses found some evidence that this did occur, but even after adjustment for confounding there was still strong evidence for an association. Residual confounding cannot be ruled out but we think it unlikely that it could explain an association of this size.

A limitation of our study is the loss to follow up from the original ALSPAC sample. The young adults were more likely to attend the clinic if they came from families of a higher education and social class, indicating that data were not missing completely at random. Non-random response may therefore have biased our complete case analyses. However, the frequency of adolescent victimisation did not differ between the original ALSPAC sample and the complete case sample, and the results of analyses after multiple imputation led to some attenuation of the findings but were consistent with our complete case findings. The wealth of data on participants from previous assessments in ALSPAC allowed for imputation of missing data using a rich list of relevant variables. The extensive auxiliary information also ensures that the assumptions behind our imputation of missing data are much more reasonable. Even though we think it unlikely that such a strong association could be explained by attrition, selection bias remains a possibility.

A second limitation is that our principal measure of victimisation was self reported and people who are prone to depression may also be more likely to perceive or report incidences of victimisation. To tackle this, we adjusted the analysis for concurrent depressive symptoms and controlled for previous victimisation and emotional and behavioural problems. Furthermore, in additional sensitivity analyses we found that the association remained even after excluding participants who scored in the clinical range for depression at age 13 years. If anything this might have led to some over-adjustment if, as we would hypothesise, depression at an earlier age had resulted from earlier victimisation. The observed association also remained when we excluded participants who at age 18 years reported experiencing depression in the clinical range for two or more years. We did not have evidence to support an association between maternally reported victimisation and depression; however, many teenagers reported that they never told anyone at home about being victimised (table 1), suggesting that mothers may not be the best informants of peer victimisation at this age.

A third limitation is that we did not specifically ask about cyberbullying (that is, bullying that takes place through the use of electronic communication). Peer victimisation was assessed in 2003-05 at a time when there was less use of electronic media. The cross sectional strength of association between peer victimisation and later depressive symptoms in childhood has been reported to be similar for cyberbullying and other peer victimisation. Furthermore, most victims report experiencing both forms.^32^ ^33^

Finally, it is not clear if our findings generalise beyond this UK population of children. Victimisation rates may vary from country to country,^34^ but peer victimisation seems to occur in all societies and we think the result of this study is likely to apply to many other societies. Furthermore, a consistent, graded association between victimisation and depressive symptoms has been observed using cross sectional data from 28 countries in Europe and North America.^35^ One study from the United States recently reported a relation between bullying in childhood and clinical depression in adulthood.^36^ We think it is likely that this result would also apply in many other societies, although such generalisability cannot be assured.

Finally, there are limitations inherent to observational data such as ours. For example, although we adjusted for several potential confounders, we cannot exclude the possibility of residual confounding.

### Victimisation in adolescence

Our findings show that victimisation in adolescence is associated with depression irrespective of previous victimisation in childhood. Adolescence is characterised by rapid social, emotional, and physiological changes. Peer relationships increase in both importance and complexity, and self consciousness is heightened. It is a critical period for the development of social relationships outside of the home and could be a particularly sensitive time if these are disrupted. Although overall rates of peer victimisation are lower in adolescence compared with childhood,^37^ evidence suggests that peer victimisation may be more targeted and persistent in the teenage years.^38^ Animal studies using models of aversive social interactions during early adolescence have reported long lasting effects, enduring into adulthood. The mechanisms underlying this could include the monoamine projections that are implicated in some theories of depression.^39^ If such mechanisms are still relevant in humans, they might also be activated in teenagers exposed to victimisation and contribute to the vulnerability to future stressors and the development of depressive symptoms. Relatively few studies have examined mediating mechanisms underlying the association between peer victimisation and depression, and it is likely that both psychosocial and biological factors play a role. Studies have suggested a potential role for perception of threat and sense of control,^40^ emotion dysregulation,^41^ blunted cortisol reactivity,^42^ and increased methylation of the serotonin transporter gene^43^ among others.

### Implications for research, practice, and policy

This study is one of the largest to date to investigate whether victimisation by peers in adolescence is associated with the emergence of clinical depression, and has dealt with some of the limitations of previous longitudinal studies. Our findings support the hypothesis that victimisation in adolescence can cause depression and could make a substantial contribution to the burden of depression in adulthood. In particular we were able to adjust for several factors that might have caused both victimisation and depression. These results accord with clinical experience in which many adults with depression report periods of bullying during adolescence. Also of relevance are the reports of suicide and self harm among adolescents attributed to victimisation, a topic of much public concern and media interest.^44^ ^45^ ^46^ Our findings also highlight that a lot of victimisation may go unreported, with many adolescents reporting that they tell no one either at home or at school.

Depression is a major public health problem worldwide, with high social and economic costs. If peer victimisation in adolescence is a causal factor then the prevention of victimisation in schools could be an effective means of reducing the incidence of depression. School based programmes based on cognitive behavioural therapy have been disappointing.^3^ Although there is evidence that consistent antibullying measures in schools can reduce the rates of victimisation,^47^ our findings suggest that such interventions during adolescence could help to reduce the burden of depression later in life.

### Research in context: systematic review

We searched PubMed for original research published in any year that prospectively investigated the impact of peer victimisation on depression meeting diagnostic criteria in late adolescence or adulthood (defined as age ≥18 years, using the terms “bullying”, “peer victimisation”, and “depression”). Most of the research in this area has focused on the short term associations between peer victimisation and depressive symptoms in childhood and was excluded. Few studies of peer victimisation used a clinically recognised measure of depression in late adolescence or adulthood. We identified only six relevant papers. Of these, one had not adjusted for key confounders, including baseline depressive symptoms,^13^ and two others had not adjusted for earlier behavioural problems^14^ ^48^ that might themselves have led to both victimisation and later depression. Two other studies^15^ ^16^ have only found statistical evidence for an association for children who are both the victims and the perpetrators of bullying. Perpetrators also display high levels of conduct problems and reactive aggression, which are themselves associated with later depression.^17^ ^18^. Finally, several studies relied on a single-item measure of peer victimisation.^14^ ^15^ ^16^ ^19^ Given the limitations of the studies identified in our literature search, it remains unclear whether peer victimisation in adolescence contributes to the overall public health burden of clinical depression.

### Interpretation

Our study is one of the largest to date to examine whether peer victimisation is prospectively associated with depression meeting diagnostic criteria in the clinically relevant range. The main strength of this study is the wide range of confounders and comprehensive measure of peer victimisation. We observed a strong, graded association of peer victimisation in adolescence with depression meeting diagnostic criteria at age 18 years, independent of the effects of the experiences of victimisation in childhood, baseline emotional and behavioural problems, and a range of other key confounders. Our findings suggest that approximately 29% of the burden of depression at age 18 years could be attributed to peer victimisation. These findings lead us to conclude that peer victimisation during adolescence may contribute significantly to the overall public health burden of clinical depression and that intervention to reduce peer victimisation in secondary schools should reduce the burden of depression.

**What is already known on this topic**

Numerous studies have shown that victimisation by peers is associated with an increased risk of internalising problems in childhood
It remains unclear whether peer victimisation contributes to the public health burden of depression that mainly manifests in adulthood because of limitations in previous studies

**What this study adds**

Our observational findings suggest that approximately 29% of the burden of depression at age 18 years could be attributed to victimisation by peers in adolescence if this relation were causal
This study used a well validated measure of peer victimisation and was able to adjust for several factors that might have caused both victimisation and depression

## Notes

We thank the families who took part in this study, the midwives for help in recruiting them, and the ALSPAC team, which includes interviewers, computer and laboratory technicians, clerical workers, research scientists, volunteers, managers, receptionists, and nurses.

Contributors: LB and GL conceived and designed the study, with advice from CJ and DW. LB analysed the data, and LB, CJ, DW and GL interpreted the data. LB drafted the manuscript. CJ, DW, and GL criticised the manuscript for important intellectual content. All authors have read and approved the final version of the manuscript. This article is the work of the authors. LB serves as guarantor for the contents of this article. All authors, external and internal, had full access to all of the data (including statistical reports and tables) in the study and can take responsibility for the integrity of the data and the accuracy of the data analysis. All researchers are independent of the funding bodies.

Ethical approval: This study was approved by the ALSPAC Law and Ethics Committee and the local research ethics committees.

Data sharing: The ALSPAC policy on data sharing is available at www.bristol.ac.uk/alspac. To discuss access to ALSPAC data, please contact the ALSPAC executive team at alspac-exec@bristol.ac.uk.

Transparency: The lead author (LB) affirms that the manuscript is an honest, accurate, and transparent account of the study being reported; that no important aspects of the study have been omitted; and that any discrepancies from the study as planned have been explained.

## Notes

Cite this as: *BMJ* 2015;350:h2469
